# 3-Benzyl­oxypyridin-2-amine

**DOI:** 10.1107/S160053680802730X

**Published:** 2008-08-30

**Authors:** De-Jun Lin, Yu Sun, Zhong-Shu Li, Bai-Wang Sun

**Affiliations:** a Ordered Matter Science Research Center, College of Chemistry and Chemical, Engineering, Southeast University, Nanjing 210096, People’s Republic of China

## Abstract

In the title compound, C_12_H_12_N_2_O, the dihedral angle between the planes of the pyridine and phenyl rings plane is 35.94 (12)°. In the crystal structure, centrosymmetrically related mol­ecules are linked by a pair of N—H⋯N hydrogen bonds, forming a dimer with an *R*_2_^2^(8) ring motif. In addition, there is an intra­molecular N—H⋯O inter­action.

## Related literature

For background, see: Sharma *et al.* (2004 ▶); Evans *et al.* (2002 ▶).
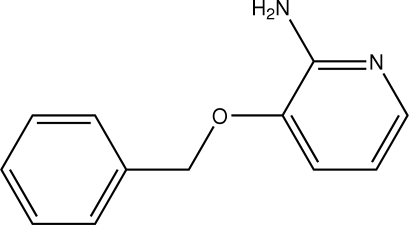


## Experimental

### 

#### Crystal data

C_12_H_12_N_2_O
*M* *_r_* = 200.24
Orthorhombic, 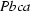

*a* = 12.852 (3) Å
*b* = 7.4068 (15) Å
*c* = 22.561 (4) Å
*V* = 2147.6 (8) Å^3^
*Z* = 8
Mo *K*α radiation
μ = 0.08 mm^−1^
*T* = 293 (2) K
0.15 × 0.10 × 0.07 mm

#### Data collection

Bruker SMART 1K CCD area-detector diffractometer
Absorption correction: multi-scan (*CrystalClear*; Rigaku, 2005 ▶) *T*_min_ = 0.982, *T*_max_ = 0.991
19827 measured reflections
2458 independent reflections
1375 reflections with *I* > 2σ(*I*)
*R*_int_ = 0.084

#### Refinement

*R*[*F* ^2^ > 2σ(*F* ^2^)] = 0.067
*wR*(*F* ^2^) = 0.157
*S* = 1.07
2458 reflections
136 parameters
H-atom parameters constrained
Δρ_max_ = 0.13 e Å^−3^
Δρ_min_ = −0.15 e Å^−3^

### 

Data collection: *CrystalClear* (Rigaku, 2005 ▶); cell refinement: *CrystalClear*; data reduction: *CrystalClear*; program(s) used to solve structure: *SHELXS97* (Sheldrick, 2008 ▶); program(s) used to refine structure: *SHELXL97* (Sheldrick, 2008 ▶); molecular graphics: *SHELXTL* (Sheldrick, 2008 ▶); software used to prepare material for publication: *SHELXTL*.

## Supplementary Material

Crystal structure: contains datablocks I, global. DOI: 10.1107/S160053680802730X/at2616sup1.cif

Structure factors: contains datablocks I. DOI: 10.1107/S160053680802730X/at2616Isup2.hkl

Additional supplementary materials:  crystallographic information; 3D view; checkCIF report

## Figures and Tables

**Table 1:** Hydrogen-bond geometry (Å, °)

*D*—H⋯*A*	*D*—H	H⋯*A*	*D*⋯*A*	*D*—H⋯*A*
N1—H1*B*⋯N2^i^	0.86	2.18	3.021 (3)	166
N1—H1*C*⋯O1	0.86	2.29	2.628 (3)	104

Symmetry code: (i) 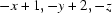.

**Fig. 1.**
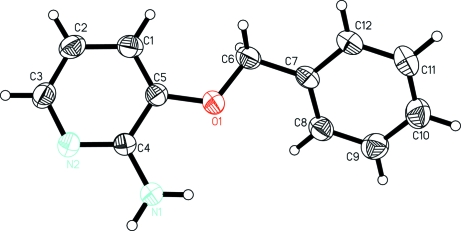
The molecular structure of the title molecule, with the atom-numbering scheme. Displacement ellipsoids are drawn at the 30% probability level. The intramolecular N—H···O contact is shown as a dashed line.

**Fig. 2.**
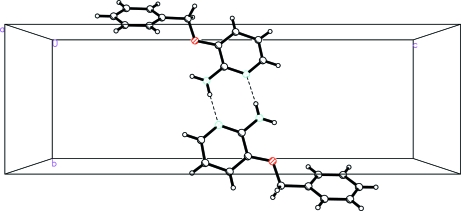
View of the packing and hydrogen bonding of the compound (I), down along the b axis. Hydrogen bonds are shown as dashed lines. H atoms not involved in hydrogen bonding have been omitted for clarity.

## supplementary crystallographic information

### Comment

Benzyl ethers and their derivatives are used as protecting group (Sharma *et al.*, 2004) for alcohols and phenols in the synthesis of natural products (Evans *et al.*, 2002). Here we report the synthesis and structure of the title compound, namely 3-(benzyloxy)pyridin-2-amine (I).

In the title compound (I) (Fig.1), the dihedral angle between the pyridine ring plane and benzene ring plane is 35.94 (12)°. In the crystal structure, centrosymmetrically related molecules are linked by a pair of N—H···N hydrogen bonds to form a dimer with an *R*_2_^2^(8) ring motif (Fig. 2). In addition, there is an intramolecular N—H···O interaction (Table 1).

### Experimental

3-(Benzyloxy)pyridin-2-amine (0.020 g, 0.1 mmol) was added to a solution containing ethanol (8 ml) and ether (4 ml). The mixture was stirred at room temperature for 10 min and then filtered off. After a few days, colourless single crystals were obtained.

### Refinement

All H atoms attached to C and N atom were fixed geometrically and treated as riding with C—H = 0.93 Å (aromatic) or 0.97 Å (methylene) and N—H = 0.86 Å, and with *U*_iso_(H) =1.2*U*_eq_(C or N).

### Crystal data

**Table:**

C_12_H_12_N_2_O	*F*_000_ = 848
*M**_r_* = 200.24	*D*_x_ = 1.239 Mg m^−^^3^
Orthorhombic, *P**b**c**a*	Mo *K*α radiation λ = 0.71073 Å
Hall symbol: -P 2ac 2ab	Cell parameters from 21430 reflections
*a* = 12.852 (3) Å	θ = 3.1–27.5º
*b* = 7.4068 (15) Å	µ = 0.08 mm^−^^1^
*c* = 22.561 (4) Å	*T* = 293 (2) K
*V* = 2147.6 (8) Å^3^	Block, colourless
*Z* = 8	0.15 × 0.10 × 0.07 mm

### Data collection

**Table:**

Bruker SMART 1K CCD area-detector diffractometer	2458 independent reflections
Radiation source: fine-focus sealed tube	1375 reflections with *I* > 2σ(*I*)
Monochromator: graphite	*R*_int_ = 0.084
Detector resolution: 8.192 pixels mm^-1^	θ_max_ = 27.5º
*T* = 293(2) K	θ_min_ = 3.2º
Thin–slice ω scans	*h* = −16→16
Absorption correction: multi-scan(CrystalClear; Rigaku, 2005)	*k* = −9→9
*T*_min_ = 0.982, *T*_max_ = 0.991	*l* = −29→29
19827 measured reflections	

### Refinement

**Table:**

Refinement on *F*^2^	Secondary atom site location: difference Fourier map
Least-squares matrix: full	Hydrogen site location: inferred from neighbouring sites
*R*[*F*^2^ > 2σ(*F*^2^)] = 0.067	H-atom parameters constrained
*wR*(*F*^2^) = 0.157	*w* = 1/[σ^2^(*F*_o_^2^) + (0.0492*P*)^2^ + 0.5758*P*] where *P* = (*F*_o_^2^ + 2*F*_c_^2^)/3
*S* = 1.07	(Δ/σ)_max_ < 0.001
2458 reflections	Δρ_max_ = 0.13 e Å^−^^3^
136 parameters	Δρ_min_ = −0.15 e Å^−^^3^
Primary atom site location: structure-invariant direct methods	Extinction correction: none

### Special details

**Table:**

Geometry. All e.s.d.'s (except the e.s.d. in the dihedral angle between two l.s. planes) are estimated using the full covariance matrix. The cell e.s.d.'s are taken into account individually in the estimation of e.s.d.'s in distances, angles and torsion angles; correlations between e.s.d.'s in cell parameters are only used when they are defined by crystal symmetry. An approximate (isotropic) treatment of cell e.s.d.'s is used for estimating e.s.d.'s involving l.s. planes.
Refinement. Refinement of *F*^2^ against ALL reflections. The weighted *R*-factor *wR* and goodness of fit *S* are based on *F*^2^, conventional *R*-factors *R* are based on *F*, with *F* set to zero for negative *F*^2^. The threshold expression of *F*^2^ > σ(*F*^2^) is used only for calculating *R*-factors(gt) *etc*. and is not relevant to the choice of reflections for refinement. *R*-factors based on *F*^2^ are statistically about twice as large as those based on *F*, and *R*- factors based on ALL data will be even larger.

### Fractional atomic coordinates and isotropic or equivalent isotropic displacement parameters (Å^2^)

**Table:**

	*x*	*y*	*z*	*U*_iso_*/*U*_eq_	
C1	0.66421 (18)	0.4734 (3)	−0.00746 (11)	0.0624 (7)	
H1A	0.6925	0.3609	0.0012	0.075*	
C2	0.65962 (19)	0.5359 (4)	−0.06581 (11)	0.0689 (7)	
H2A	0.6860	0.4667	−0.0967	0.083*	
C3	0.61591 (19)	0.6997 (4)	−0.07666 (11)	0.0648 (7)	
H3A	0.6124	0.7391	−0.1157	0.078*	
C4	0.58293 (17)	0.7505 (3)	0.02173 (10)	0.0501 (6)	
C5	0.62616 (17)	0.5814 (3)	0.03648 (10)	0.0502 (6)	
C6	0.65634 (19)	0.3704 (3)	0.11541 (11)	0.0630 (7)	
H6A	0.7283	0.3488	0.1046	0.076*	
H6B	0.6135	0.2785	0.0969	0.076*	
C7	0.64405 (17)	0.3635 (3)	0.18162 (11)	0.0546 (6)	
C8	0.5582 (2)	0.4375 (3)	0.20964 (11)	0.0659 (7)	
H8A	0.5056	0.4899	0.1871	0.079*	
C9	0.5499 (2)	0.4343 (4)	0.27071 (12)	0.0763 (8)	
H9A	0.4922	0.4855	0.2891	0.092*	
C10	0.6265 (3)	0.3558 (4)	0.30437 (13)	0.0833 (9)	
H10A	0.6213	0.3540	0.3455	0.100*	
C11	0.7103 (3)	0.2807 (4)	0.27693 (13)	0.0899 (9)	
H11A	0.7617	0.2256	0.2996	0.108*	
C12	0.7201 (2)	0.2850 (3)	0.21579 (12)	0.0726 (8)	
H12A	0.7784	0.2346	0.1978	0.087*	
N1	0.54785 (16)	0.8603 (3)	0.06572 (8)	0.0714 (7)	
H1B	0.5227	0.9648	0.0572	0.086*	
H1C	0.5508	0.8253	0.1020	0.086*	
N2	0.57740 (15)	0.8083 (3)	−0.03408 (8)	0.0577 (5)	
O1	0.62485 (13)	0.5449 (2)	0.09615 (7)	0.0628 (5)	

### Atomic displacement parameters (Å^2^)

**Table:**

	*U*^11^	*U*^22^	*U*^33^	*U*^12^	*U*^13^	*U*^23^
C1	0.0613 (16)	0.0619 (15)	0.0641 (17)	0.0114 (13)	−0.0064 (12)	−0.0070 (13)
C2	0.0723 (17)	0.0770 (18)	0.0575 (17)	0.0122 (14)	0.0023 (13)	−0.0152 (14)
C3	0.0654 (16)	0.0796 (18)	0.0494 (14)	0.0048 (14)	0.0042 (12)	−0.0011 (13)
C4	0.0492 (13)	0.0506 (13)	0.0506 (13)	0.0023 (11)	0.0016 (10)	0.0002 (11)
C5	0.0462 (13)	0.0560 (14)	0.0484 (13)	0.0015 (10)	−0.0037 (10)	−0.0016 (11)
C6	0.0676 (17)	0.0540 (15)	0.0673 (17)	0.0115 (12)	−0.0057 (13)	0.0004 (12)
C7	0.0581 (15)	0.0411 (12)	0.0645 (15)	0.0025 (11)	−0.0048 (12)	0.0068 (11)
C8	0.0588 (15)	0.0680 (16)	0.0709 (17)	0.0074 (13)	−0.0004 (13)	0.0095 (13)
C9	0.0757 (19)	0.0759 (19)	0.077 (2)	0.0016 (15)	0.0155 (15)	0.0037 (15)
C10	0.105 (2)	0.079 (2)	0.0653 (18)	0.0010 (18)	0.0042 (17)	0.0146 (16)
C11	0.105 (3)	0.091 (2)	0.074 (2)	0.0223 (19)	−0.0172 (18)	0.0197 (17)
C12	0.0774 (19)	0.0659 (17)	0.0747 (18)	0.0221 (14)	−0.0066 (14)	0.0062 (14)
N1	0.1049 (18)	0.0599 (12)	0.0493 (11)	0.0246 (12)	0.0085 (11)	0.0024 (10)
N2	0.0612 (13)	0.0633 (12)	0.0486 (11)	0.0010 (10)	0.0049 (9)	0.0027 (10)
O1	0.0838 (12)	0.0531 (10)	0.0517 (10)	0.0124 (8)	−0.0015 (8)	0.0041 (8)

### Geometric parameters (Å, °)

**Table:**

C1—C5	1.365 (3)	C6—H6B	0.9700
C1—C2	1.397 (3)	C7—C12	1.374 (3)
C1—H1A	0.9300	C7—C8	1.384 (3)
C2—C3	1.359 (3)	C8—C9	1.382 (3)
C2—H2A	0.9300	C8—H8A	0.9300
C3—N2	1.347 (3)	C9—C10	1.373 (4)
C3—H3A	0.9300	C9—H9A	0.9300
C4—N2	1.332 (3)	C10—C11	1.361 (4)
C4—N1	1.360 (3)	C10—H10A	0.9300
C4—C5	1.410 (3)	C11—C12	1.386 (4)
C5—O1	1.373 (3)	C11—H11A	0.9300
C6—O1	1.422 (3)	C12—H12A	0.9300
C6—C7	1.503 (3)	N1—H1B	0.8600
C6—H6A	0.9700	N1—H1C	0.8600
			
C5—C1—C2	118.4 (2)	C12—C7—C6	119.8 (2)
C5—C1—H1A	120.8	C8—C7—C6	121.6 (2)
C2—C1—H1A	120.8	C9—C8—C7	120.7 (2)
C3—C2—C1	118.9 (2)	C9—C8—H8A	119.7
C3—C2—H2A	120.5	C7—C8—H8A	119.7
C1—C2—H2A	120.5	C10—C9—C8	120.2 (3)
N2—C3—C2	123.8 (2)	C10—C9—H9A	119.9
N2—C3—H3A	118.1	C8—C9—H9A	119.9
C2—C3—H3A	118.1	C11—C10—C9	119.3 (3)
N2—C4—N1	118.7 (2)	C11—C10—H10A	120.4
N2—C4—C5	122.0 (2)	C9—C10—H10A	120.4
N1—C4—C5	119.3 (2)	C10—C11—C12	121.1 (3)
C1—C5—O1	127.0 (2)	C10—C11—H11A	119.5
C1—C5—C4	119.4 (2)	C12—C11—H11A	119.5
O1—C5—C4	113.67 (19)	C7—C12—C11	120.2 (3)
O1—C6—C7	107.75 (18)	C7—C12—H12A	119.9
O1—C6—H6A	110.2	C11—C12—H12A	119.9
C7—C6—H6A	110.2	C4—N1—H1B	120.0
O1—C6—H6B	110.2	C4—N1—H1C	120.0
C7—C6—H6B	110.2	H1B—N1—H1C	120.0
H6A—C6—H6B	108.5	C4—N2—C3	117.5 (2)
C12—C7—C8	118.6 (2)	C5—O1—C6	118.36 (18)
			
C5—C1—C2—C3	−1.2 (4)	C7—C8—C9—C10	−0.6 (4)
C1—C2—C3—N2	0.9 (4)	C8—C9—C10—C11	−0.3 (4)
C2—C1—C5—O1	−179.2 (2)	C9—C10—C11—C12	1.1 (5)
C2—C1—C5—C4	0.6 (3)	C8—C7—C12—C11	0.0 (4)
N2—C4—C5—C1	0.4 (3)	C6—C7—C12—C11	178.9 (2)
N1—C4—C5—C1	−178.1 (2)	C10—C11—C12—C7	−1.0 (5)
N2—C4—C5—O1	−179.8 (2)	N1—C4—N2—C3	177.8 (2)
N1—C4—C5—O1	1.7 (3)	C5—C4—N2—C3	−0.7 (3)
O1—C6—C7—C12	−137.2 (2)	C2—C3—N2—C4	0.0 (4)
O1—C6—C7—C8	41.6 (3)	C1—C5—O1—C6	−6.8 (3)
C12—C7—C8—C9	0.7 (4)	C4—C5—O1—C6	173.45 (19)
C6—C7—C8—C9	−178.1 (2)	C7—C6—O1—C5	−177.93 (18)

### Hydrogen-bond geometry (Å, °)

**Table:**

*D*—H···*A*	*D*—H	H···*A*	*D*···*A*	*D*—H···*A*
N1—H1B···N2^i^	0.86	2.18	3.021 (3)	166
N1—H1C···O1	0.86	2.29	2.628 (3)	104

Symmetry codes: (i) −*x*+1, −*y*+2, −*z*.
